# Genome Assembly of Azotobacter chroococcum Strain W5, a Free-Living Diazotroph Isolated from India

**DOI:** 10.1128/MRA.00259-20

**Published:** 2020-05-14

**Authors:** Annapurna Kannepalli, Kedharnath Reddy Pengani, Jeshima Khan Yasin, Sangeeta Paul, Swati Tyagi, Govindasamy Venkadasamy, Meenakshi Sharma, Swarnalakshmi Karivaradharajan

**Affiliations:** a Division of Microbiology, ICAR-Indian Agricultural Research Institute, New Delhi, India; b Division of Genomic Resources, ICAR-NBPGR, PUSA Campus, New Delhi, India; Broad Institute

## Abstract

Azotobacter chroococcum strain W5 (MTCC 25045) is an effective diazotrophic bacterium with plant growth-promoting traits. Here, we report the draft genome assembly of this biologically and agronomically evaluated *A. chroococcum* strain. The genome assembly in 55 contigs is 4,617,864 bp long, with a G+C content of 66.83%.

## ANNOUNCEMENT

*Azotobacter* spp. are Gram-negative, free-living, aerobic, soil-dwelling, oval or spherical bacteria that form thick-walled cysts (as a means of asexual reproduction under favorable conditions). There are seven known species of the genus.

A Gram-negative, aerobic, nitrogen-fixing bacterium was isolated from the rhizosphere of wheat (Triticum aestivum L.) grown at Indian Agricultural Research Institute (IARI) research farms and identified as Azotobacter chroococcum based on 16S rRNA gene sequencing (GenBank accession no. MT299751). The bacterium grew luxuriously on N_2_-free mineral medium and produced brown pigment and plant growth-promoting hormones (1). This isolate was named Azotobacter chroococcum strain W5 (MTCC 25045). Inoculation with *A. chroococcum* strain W5 led to an increase in crop yield (2, 3). These ﬁndings prompted further efforts to investigate this bacterium for potential applications in agricultural and pharmaceutical industries.

To identify the specific genomic features of this special strain, we attempted whole-genome sequencing. Genomic DNA (gDNA) was extracted as reported earlier (4) from a 72-h culture grown in Luria-Bertani medium that was inoculated with a single isolated colony using a standard isolation protocol as per the manufacturer’s instructions (Qiagen DNA extraction kit). The integrity and quality of the DNA were checked through a fluorescence-based quantitation assay as per the developer’s instructions (Qubit 3.0; Thermo Fisher). An ultrasonicator (Covaris) was used to shear the genomic DNA and to generate 350-bp-long fragments. DNA libraries (NEBNext Ultra DNA library prep kit; Illumina, USA) were generated following the manufacturer’s recommendations. The fragments were end polished, A tailed, and ligated to full-length adapters for Illumina sequencing. In the final preparation step, PCR products were purified (AMPure XP system). Libraries were analyzed for size distribution using a Bioanalyzer (Agilent 2100) (5) and quantified using real-time PCR (CFX96; Bio-Rad). Illumina paired-end sequencing (HiSeq 1500) resulted in 14,040,301 raw reads with a read length of 150 bases, totaling 4.2 GB of data output. The quality of the reads generated was assessed using FastQC v0.11.5 (6) (https://www.bioinformatics.babraham.ac.uk/projects/fastqc/). Of the total raw reads generated, 14.24% were below a Phred score of 40. Raw reads with N (unidentified base) and Phred scores less than 30 were filtered out using Trimmomatic v0.39 (7, 8) before further analyses with Illumina adapter sequences (ATCACG) (9). The high-quality clean data of 3.6 Gb with 13,178,049 reads were subjected to genome assembly using SPAdes v3.11.1 with default settings (10). Table 1 displays the assembly details of Azotobacter chroococcum strain W5.

**TABLE 1:** Azotobacter chroococcum strain W5 sequencing and assembly metrics

Genome feature	Value(s)
Sequence length (bp)	4,617,864
No. of contigs	55
G+C content (%)	66.83
Shortest contig length (bp)	248
Longest contig length (bp)	476,631
No. of repeat regions	1
Total no. of genes	4,335
Total no. of CDSs^*a*^	4,273
Total no. of coding genes	4,168
No. of tRNAs	53
No. of rRNAs	5 (3 5S, 1 16S, and 1 23S)
No. of ncRNAs	4
No. of CRISPRs	1
*N*_50_ (bp)	257,772
Total no. of pseudogenes	105

a CDSs, coding DNA sequences.

The smallest and largest contig sizes were 248 and 476,631 bases, respectively. Finally, 55 contigs with an *N*_50_ value of 257,772 bases were assembled into the genome of 4,617,864 bases, coding 4,335 genes and 105 pseudogenes. Furthermore, 53 tRNAs, 5 rRNAs, and 4 noncoding RNAs (ncRNAs) were predicted by using the NCBI Prokaryotic Genome Annotation Pipeline (PGAP) v4.11 (11). Overall, the G+C content of the assembly was 66.83%. Siderophore- and bacteriocin-coding genes were predicted by antiSMASH v5.0.0 using default settings (12).

### Data availability.

The complete genome sequence of *A. chroococcum* strain W5 has been deposited in DDBJ/ENA/GenBank under the accession no. JAAPAP000000000 (BioProject no. PRJNA610299, BioSample no. SAMN14292004, and SRA accession no. SRR11237782). The version described in this paper is the first version, JAAPAP010000000.
